# Reflecting on a decade of action: a review of select indicators for the Israel National Program for Active and Healthy Living–Efsharibari, 2011–2022

**DOI:** 10.1186/s13584-025-00722-3

**Published:** 2025-10-22

**Authors:** Sharon Levi, Liri Endy Findling, Ronit Endevelt, Efrat Aflalo

**Affiliations:** 1 Department of Health Systems Management, Max Stern Yezreel Valley College, Mizra, Yezreel Valley, Israel; 2 Israel National Program for Active and Healthy Living – Efsharibari, Ministry of Health, Jerusalem, Israel; 3 Health Education and Promotion Department, Public Health Directorate, Ministry of Health, Jerusalem, Israel; 4 School of Public Health, University of Haifa, Haifa, Israel; 5 Nutrition Division, Public Health Directorate, Ministry of Health, Jerusalem, Israel; 6 Physicians and Required Medical Professions Directorate, Ministry of Aliyah and Integration, Jerusalem, Israel

**Keywords:** Health policy, National program, Obesity, Physical activity, Indicators, Evaluation

## Abstract

**Background:**

Similar to countries worldwide, Israel suffers from the effects of a modern lifestyle, with high rates of obesity, lack of healthy nutrition and insufficient physical activity. Global health organizations recommend national systemic programs to address this problem. The Israel National Program for Active and Healthy Living–Efsharibari was launched at the end of 2011 via government resolution, to reduce morbidity and mortality and improve quality of life for the diverse population. It is a collaborative effort of the Ministries of Health, Culture and Sport, and Education.

**Methods:**

Development and monitoring of a comprehensive set of indicators to assess process, outputs and outcomes in relation to program objectives. Qualitative and quantitative retrospective review of legislation, standards and health-promoting environments. Review of national data and surveys related to key activity domains – policy, supportive environments and social marketing – and a study of related behavioral and morbidity indicators.

**Results:**

Over a decade, notable changes were observed for indicators in key activity domains, aligning with the programs’ initial objectives. Improvements were found for 13 of 18 planned nutrition policies and 7 of 8 physical-activity policies. Changes to the nutritional environment included enhanced nutritional labeling and reductions in the amounts of sodium, sugar and saturated fat in commercial food products. An increase in national and local health promotion infrastructures included adoption of the “healthy city” protocol in 77 local authorities and implementation of the “health-promoting school” plan in 1,698 schools across the country. Behavior and morbidity indicators display mixed findings: reported behaviors in the field of nutrition have improved and physical activity among adults 65 + has increased. However, fewer children, youth, and adults are achieving the global recommended levels of reported physical activity and there are negative trends in breastfeeding behaviors. In terms of morbidity, a decrease in mortality from heart disease and diabetes was found, along with modest fluctuations in obesity trends.

**Conclusions:**

Consistent and ongoing efforts of the national plan to promote health in all policies, enable healthy choices, and promote systematic change have impacted Israeli society over time. In spite of changes of government and a lack of funds, Efsharibari has allowed for continuous cooperation between policymakers, professionals and additional partners, including during the COVID-19 pandemic. Additional efforts are necessary to reduce health disparities, enhance healthy behaviors and improve morbidity indicators. Amidst ongoing international dialogue to improve and expand health promotion, Israel’s practical experience, including both successes and challenges serves to inform the international community.

**Supplementary Information:**

The online version contains supplementary material available at 10.1186/s13584-025-00722-3.

## Background

Noncommunicable diseases (NCDs), including heart disease, cancer, and diabetes, are a leading cause of morbidity and mortality worldwide [1]. In Israel in recent decades NCDs are the cause of 85% of deaths. Israel also has substantial rates of obesity and low levels of physical activity [2].

Lack of a healthy lifestyle is a significant factor for risk of NCDs. Behaviors, including physical activity, healthy nutrition, reduced smoking and reduced alcohol consumption are related to reductions in morbidity and mortality [3, 4]. Physical activity has been found to be beneficial in reducing the risk of disease, anxiety and depression as well as improving cognitive function, muscle and skeletal health and sleep function [4–6]. A balanced diet has been found to be effective in reducing the risk of chronic disease, while the consumption of processed foods (rich in sodium, saturated fat and sugar) contribute significantly to increased rates of cardiovascular diseases, cancer and diabetes [7–9]. Social determinants and socio-economic characteristics such as employment, education, income and community have a major impact on health and a healthy lifestyle. As well as environmental factors, such as the quality of drinking water, clean air and amenities such as parks and walking paths [10, 11].

The global action plan for the prevention and control of NCDs recommends a “health in all policies” (HiAP) approach, with an emphasis on leadership and multi-sector collaborations at the national and regional levels [12]. Moreover, comprehensive government policies including a variety of complementary interventions are recommended [4].

### Initiation of the Israel National Program for Active and Healthy Living–Efsharibari

In December 2011, decision HC/237 was passed and the Israeli government launched the Israel National Program for Active and Healthy Living–Efsharibari (Efsharibari in Hebrew means "A healthy lifestyle is possible") as a response to morbidity and mortality rates due to NCDs [13].

Efsharibari is a joint initiative of the MOH, the Ministry of Culture and Sports and the Ministry of Education. Since its inception, an inter-ministerial steering committee has led the program as ‘a joint national effort to promote an active and healthy life’, with an emphasis on the promotion of healthy nutrition and physical activity [13]. Over the course of the first decade, several focus areas were added to the Efsharibari framework, including a smoke-free environment, healthy sleep and screen habits and increased personal and community resilience. Efsharibari targets physical health and wellbeing across diverse populations and settings [13]. Efsharibari is included in the MOH prevention and health-promotion master plan and in the Healthy Future 2020 goals to improve the quality of life and extend the life expectancy of the population, while reducing disparities [14].

Efsharibari uses health promotion strategies and promotes action areas in line with the Ottawa Charter for Health Promotion [15], including policy and legislation, supporting environments, community programming, and social marketing. Specific program actions and objectives include: (i) Plan, develop and implement policies that support a healthy lifestyle; (ii) Develop an environment that enables and supports health – physically, socially, and financially; (iii) Develop and maintain a network of connections, initiatives and partnerships between ministries and stakeholders; and (iv) Make active and healthy living a social norm [13].

At the inception of Efsharibari, overarching aims were defined including: increasing physical activity, increasing healthy nutrition, decreasing unhealthy nutrition; and reducing obesity rates. These aims were categorized into a series of objectives and indicators to monitor and evaluate progress. Periodic annual reports provided information on progress e.g. [16]. The indicators have been updated over the years in accordance with international recommendations, as well as due to limitations and changes in measurement methods. The aim of the current study was to conduct a review of select indicators at the conclusion of a decade of action to identify achievements and highlight gaps for continued implementation of the Efsharibari program. The study included a qualitative and quantitative review of process, output and outcome indicators representing the key activity domains.

## Methods

### Evaluation model

**Fig. 1:**
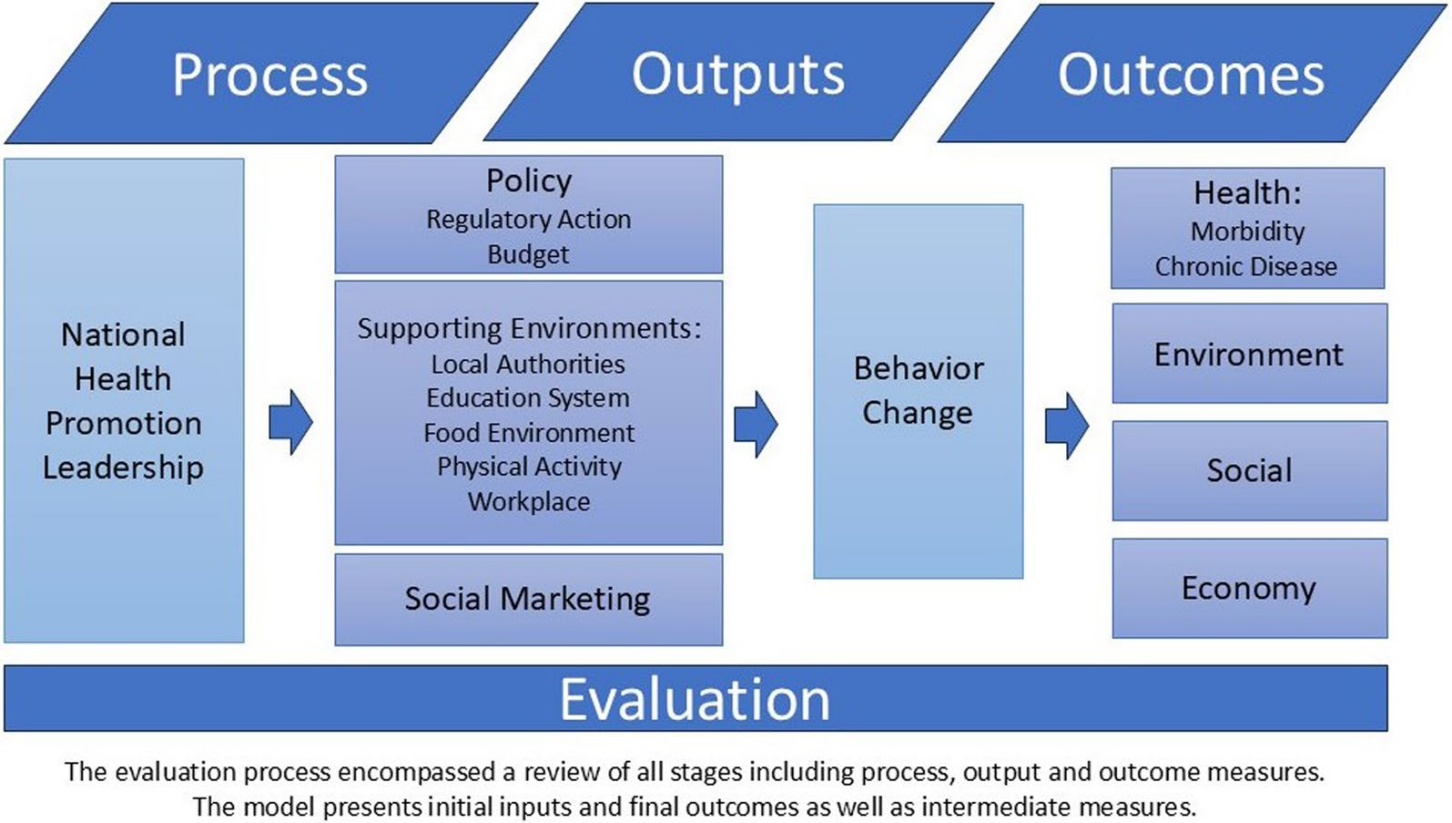
Israel national program for active and healthy living–efsharibari logic model

The Efsharibari evaluation model was developed at program inception by the inter-ministerial steering committee. The steering committee received guidance from a scientific committee including representatives from government ministries, five universities, representatives of the Israeli Center for Disease Control (ICDC) and other research institutes. It was influenced by a review of recommendations and academic literature and it took into account the availability of data sources in Israel. The basis is a logic model developed by the World Health Organization (WHO) for assessment and measurement [17]. The aim of the model was to develop a comprehensive set of indicators to systematically monitor process, outputs and outcomes of the national program over time related to key activity domains: national leadership and policy, supporting environments, and social marketing as well as related behavioral and morbidity measures (See Fig. 1). The scope of the original indicator set was very large, including 330 indices divided into 37 groups using both national and local level data based on a variety of information sources.

### Process for reviewing indicator set

The research team assessed national data and findings for the different activity domains at several points in time, before, during and at the end of the decade. A focused retrospective review of key indicators was undertaken July 2022-July 2023 to characterize a decade of health promotion within the Efsharibari program. In order to focus the assessment process and enable the identification of trends, the research team selected groups of indicators, inclusion was based on relevance and the available information sources. Indicators were excluded following a review by the research team due to repetition, lesser relevance or lack of timely data. The selected indicators presented in the results section were assessed to review change over the decade from the launch of Efsharibari. This descriptive process included an examination of published documents and the data collected throughout the time period. Based on data availability, the pre-measurement included indicators from one of the years between 2009 and 2012. The final measure included indicators collected for one of the years between 2019 and 2022, or from the last available publication year. Where possible, indicators were also collected during intermediate years. Findings for the two outer datapoints are presented throughout the manuscript; details for the complete schedule of measurements is presented in a Supplemental Table.

The analysis and presentation of trends and change over time for the key indicators, including output and outcome measures, is dependent on the activity domain, according to the following procedures.

1. **Policy domain**–progress assessment for each policy output measure, identified in the initial program objectives, is based on documentation and interviews with key agency representatives including: Director and Deputy Director, Department of Education and Health Promotion; Director Nutrition Division, Ministry of Health; Supervisor of the Health Sector, Supervisor of the Physical Education Sector, National Health Education Instructors, Ministry of Education; Deputy Head of the Sports Directorate, Director of the Department of Women’s Sports and Popular Sports at the Ministry of Culture and Sports. The interviews followed a semi-structured protocol. Participants reviewed and assessed the status of each policy. That included designating whether the policy was on the agenda, adopted, or incorporated in an action plan. Further discussions reviewed national leadership, supporting budget and resources within the program. The interviews were conducted by two members of the research team. Based on findings from the interviews and a review of relevant documents a designated scale of 1–5 was used, wherein 1 = no progress, 2 = issue identified and put on the agenda, 3 = policy clearly stated and adopted, 4 = policy is incorporated into an action plan, and 5 = policy is enforced and/or controlled. The designation was initially determined by one member of the team, and reviewed by the rest of the team. The rating scale initially developed in 2017 was evaluated for content validity by a team, the scale was revisited during the current study to assess validity for the study. Statistical tests of validity were not conducted. The findings on national leadership and the budget are presented descriptively within the policy domain.
2. **Supporting environments**–progress assessment for output measures in the various environments and health -promoting settings as detailed in the Supplemental Table. These include measuring the involvement level of local authorities and in the education system. These also include measures of access to healthy food and access to facilities and training for physical activity. The assessment comprises a qualitative review of program documentation as well as a quantitative descriptive review of measures of change, such as the increase in health-promoting settings. The quantitative data is based on multiple data sources that were reviewed systematically by members of the research team.
3. **Social marketing**–descriptive review of progress for this output measure is based on three key areas: brand implementation, digital media and national campaigns. All members of the research team reviewed survey data, media channel users (based on media monitoring tools), viewership and campaign activity. Based on the available data the research team discussed and presented key results.
4. **Health behaviors and morbidity** – descriptive assessment of outcome measures includes a comparison of initial and final quantitative data to track positive, negative or neutral trends. The datasets that were included are large-scale nationally representative surveys or national datasets inclusive of a large percentage of the population as detailed in the Supplemental Table.

## Results

The analysis identifies whether changes have been in accordance with program objectives during the first decade of Efsharibari. These objectives include healthier nutrition, increased physical activity, fostering leadership, advocacy and the development of national and local infrastructures in support of a health-promoting environment. Findings for health behaviors, morbidity and mortality are mixed. Tables for each of the activity domains present findings, using the following legend: Green–trends in indicators in the direction that is intuitively consistent with the promotion of a healthier lifestyle; Red–trends contrary to healthier lifestyles; Blue – no consistent trend; Grey – missing information. For the policy domain the findings are reported based on the designated policy scale (1–5). [–]. For other quantitative findings ± symbols present the direction of the findings. Key findings are discussed in further detail in the text. The activity domains are: policy, supporting environments, social marketing, and health behaviors and morbidity.

### Policy domain

The first decade of Efsharibari demonstrates progress for most of the policy indicators. These include substantial improvements in 13 of 18 nutrition policies and in 7 of 8 physical activity policies (see Table 1). A regulatory c ommittee including local and international nutrition and epidemiology experts was established to propose and enact nutrition policy legislation. To support the selected policies for implementation, the committee conducted several focus groups with selected populations, and conducted a participatory process by inviting the public to express their views on nutrition policy via a dedicated website [18]. Leading policies in nutrition include: a national policy to reduce sodium consumption, which was expanded in front-of-package (FOP) labeling to reduce the consumption of foods with high sodium, sugar and/or saturated fat; taxation of sweetened beverages; and reduced tax for businesses that use seasonal vegetables and fruits as refreshments. Policies with limited success include reduced pricing of foods with a high nutritional value and prohibition of marketing and advertising of foods with low nutritional value. Policies implemented to increase physical activity include the development of a national plan for physical-activity facilities plan and a policy to open, for use by the general public, the sports facilities located in schools, public buildings or public areas.

**Table 1:**
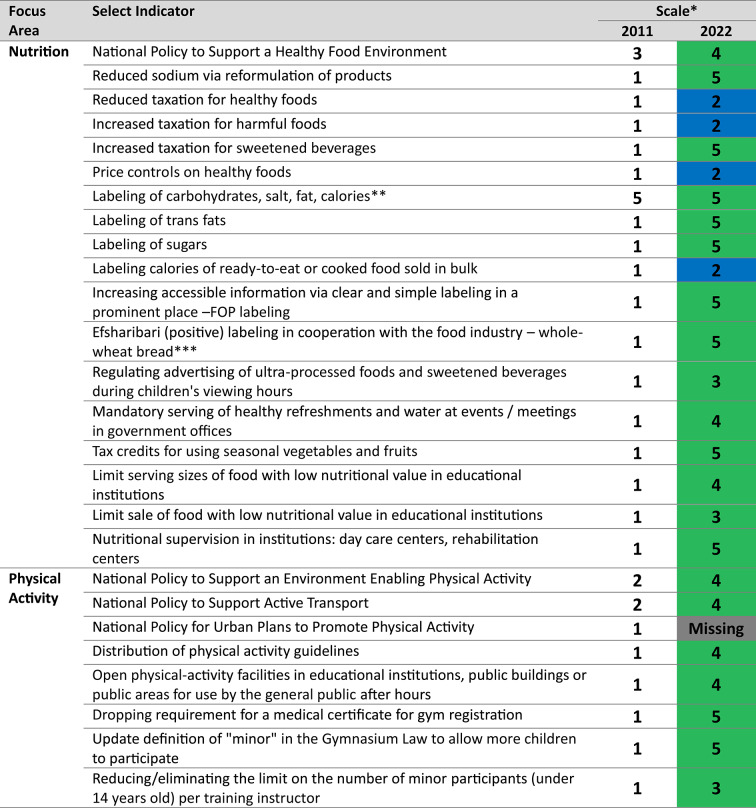
Results policy domain indicators, by focus area, 2011–2022

*Policy Scale: 1 = no progress, 2 = issue identified and put on the agenda, 3 = policy clearly stated and adopted, 4 = policy is incorporated into an action plan, and 5 = policy is enforced and/or controlled; Green indicates positive change to 3, 4 or 5 in policy scale, blue indicates little change in the policy scale, grey indicates missing information. Missing indicates that follow up information on this policy was unavailable via documentation and interviews with key agency representatives

The first decade of Efsharibari was also devoted to developing national leadership, management and coordination mechanisms to support health promotion and NCD prevention in Israel. Significant actions include the establishment of the aforementioned inter-ministerial steering committee and publication of a national strategic plan for the promotion of active and healthy living. The plan specifies a vision, goals, objectives, strategies and performance indicators [13]. Efsharibari leadership together with the inter-ministerial team developed national action plans and policies to promote active and healthy living in local jurisdictions, in the education system and at workplaces. They also developed programs and policies to help the public remain active, healthy and resilient during times of emergency, including the COVID-19 pandemic and national security situations. There have been investments in research and evaluation, including an increase in the number and scope of national surveys and research tools and participation in international measurement processes.

The budget for Efsharibari fluctuated significantly during the first decade. Initially, in 2011, the government decision included a 5-year budget of 20 million NIS (~ $5.7 million) annually, to be added to the Department of Health Education and Promotion in the MOH. This addition was to be integrated in due course into the MOH base operating budget as mandatory funds, thereby ensuring budgetary stability. This would also allow for a wider scale and long-term activities including investments in infrastructure and manpower. In practice, the planned budget was reduced from 2013 to 2015 to 2.5 million NIS (~ $715,000) discretionary funding annually. Subsequently, planned budgets continued to significantly decrease each year until 2021. The budget for Efsharibari was not integrated into the base mandatory operating budget. In 2022, discretionary funding rebounded to 20 million NIS (~ $5.7 million); however, much of this was committed to funding programming in municipalities. Partnering ministries committed relatively limited resources within the Efsharibari program. The Ministry of Education budget was 3 million NIS (~ $860,000) match from 2012 onwards, increasing to 5 million NIS (~ $1.4 million) in 2022 for health promotion programming across the education system including the Health Promoting Schools network. Initially, the Ministry of Culture and Sports provided a targeted budget for local authorities together with the MOH; however ultimately the Ministry of Culture and Sports allocated direct funding via local sports plans and its contribution to the Efsharibari budget was limited to an annual joint social marketing campaign for ‘National Walking Day’. Nonetheless, the Ministry of Culture and Sports’ funding for sports initiatives, with an emphasis on non-competitive sports, increased considerably during the decade from 2.5 to 22 million NIS (~ $715,000–6.3 million) annually.

### Supporting environments

A key activity domain emphasized in Efsharibari is the development of environments that enable and support a healthy lifestyle including physical, social, and organizational settings. Accordingly, various indicators were defined in the fields of nutrition and physical activity, as well as in key settings, such as municipalities and the education system. Table 2 details changes in the different supporting environments over a decade of action.

**Table 2:**
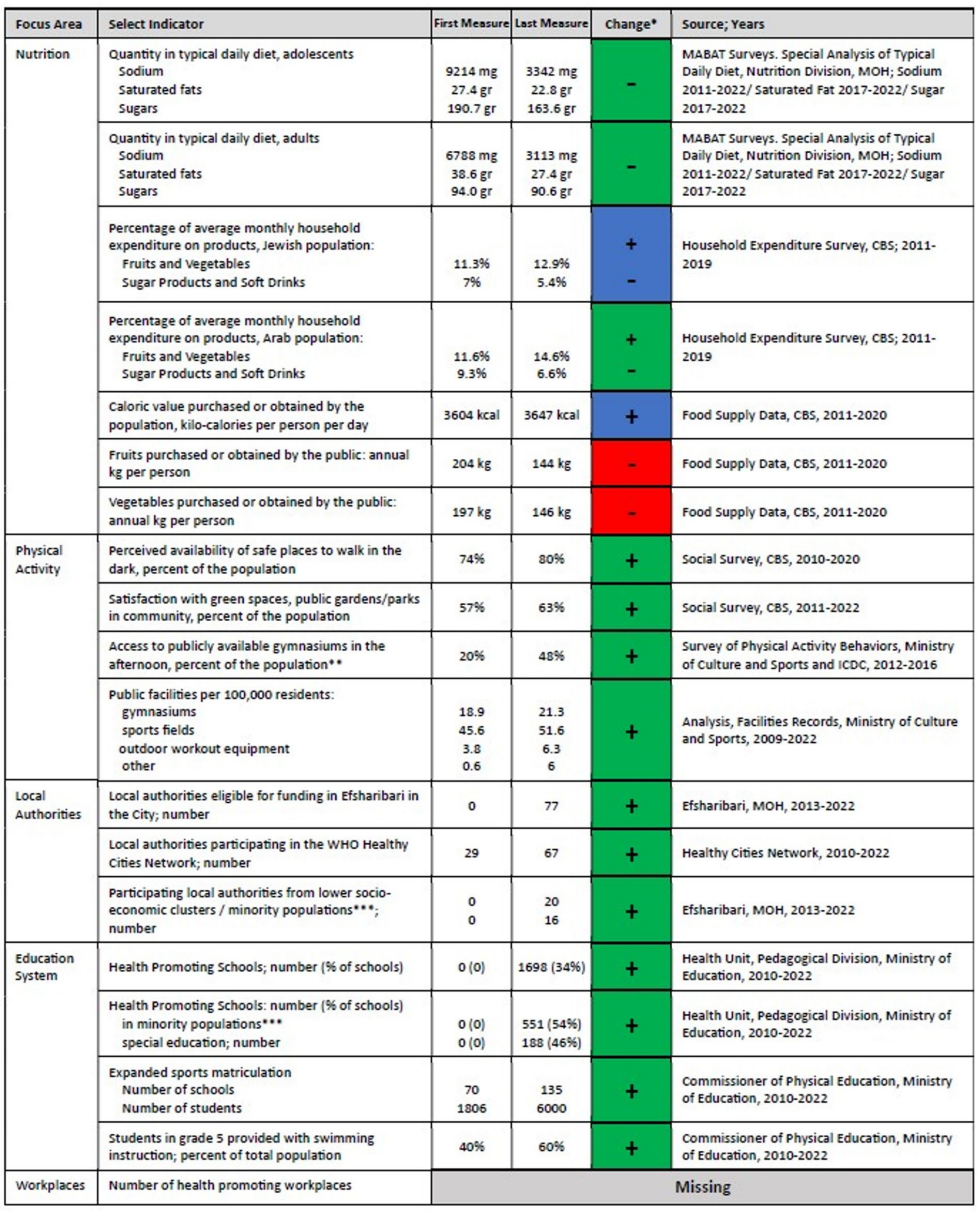
Change in supporting environment domain indicators, by focus area

* Green indicates positive change (over 2 percentage points or equivalent), red indicates negative change (over 2 percentage points or equivalent), blue indicates little change (less than 2 percentage points or equivalent), grey indicates missing information; the ± presents the direction of the findings

**Fig. 2:**
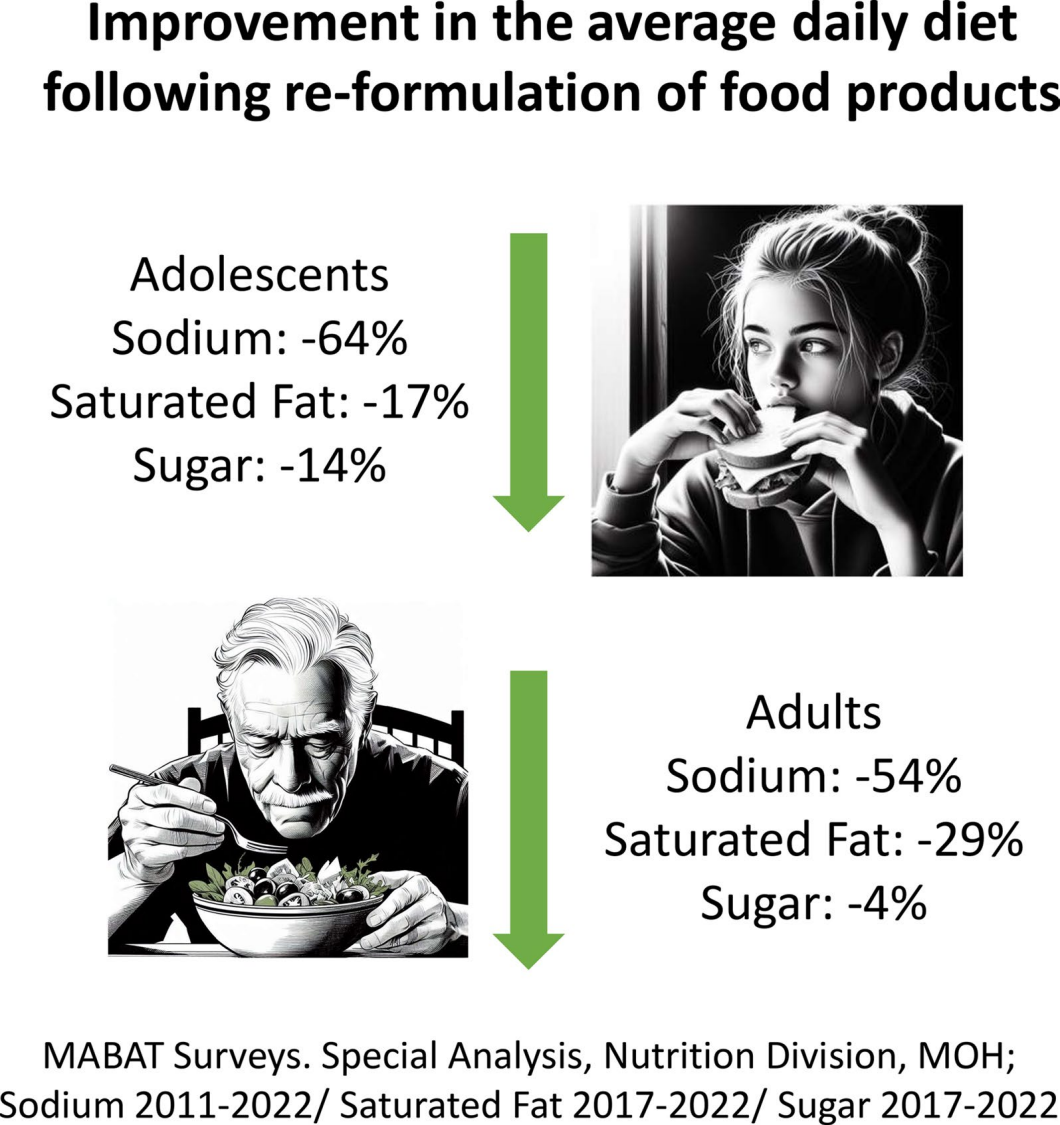
Changes in Sodium, saturated fat and sugar intake in daily diet, adolescents and adults

In the nutrition field, an offshoot of the sodium reduction and FOP labeling policies was an extensive reformulation process undertaken by food and beverage companies [19]. This commenced as a voluntary process. By 2015 over three quarters (51 of 65 companies) took part in the reformulation of common foods. Subsequently, the reformulation and labeling process resulted in a healthier nutritional environment with both adults and adolescents consuming considerably lower rates of sodium, saturated fats and sugars in their typical diet, based on the nutritional value profile for foods in the typical diet each year without requiring a change in behavior (see Table 2 and Fig. 2). Central Bureau of Statistics (CBS) data on household food expenditure paint a mixed picture, there were slight increases in the relative household expenditure on fruits and vegetables alongside a small reduction in sugar products and soft drinks, with more pronounced positive trends for the Arab population, however this may reflect changes in the cost of living rather than changes in consumption. Analysis of availability of fruits and vegetables to the public point to negative trends.

Physical activity indicators include accessibility to infrastructure and facilities that support an active lifestyle (Table 2). Positive trends were observed for several indicators. Reported access to safe places for walking in the dark and to green areas and parks increased across the population, however the percentage of those reporting that they are satisfied with the amount of green space and parks among the Jewish population is 3.2 times greater than among the Arab population, and the gaps between the Arab and Jewish populations remained the same and did not decrease during the decade. There is also an increase in most public physical activity facilities, such as sports fields and outdoor exercise equipment, as well as an increase in reported access to publicly available gymnasiums.

One of the main goals of Efsharibari at the national level was to promote health in municipalities. International experience has strongly supporting actions at the local level – e.g., the important role of local authorities in the COVID-19 pandemic [20, 21]. The WHO Healthy Cities Network, developed in the WHO European Region, aims to create healthier urban settings that support the health and well-being of the people residing in those municipalities via political leadership and commitment, changes in policy, institutions and the environment and intersectoral partnerships [22]. The network was introduced in Israel in 1990 and the MOH provided a budget to support the network from its inception. In 2012 a pilot program was designed with inter-ministerial support to promote health in municipalities with an emphasis on physical activity and nutrition. Based on the findings of the pilot, a model for a national intervention program to promote health in local authorities was developed, called ‘Efsharibari in the City’, to promote active and healthy living in municipalities. The model included: standards for a health unit including a municipal health promotion coordinator; equitable tools for financial support for activities via an annual grant cycle; and guidelines and training at the national and regional level in cooperation with the Healthy Cities Network. Accordingly, with the initiation of the ‘Efsharbari in the City’ model, there was an increase in local authorities eligible for funding each year. The Healthy Cities Network serves as the starting point for most of the cities that then proceed to participate in Efsharibari to receive funding. The two programs collaborate extensively including developing and conducting training programs for city employees, issuing white papers on health promotion in municipalities and joint research efforts. By 2022 about a third of the municipalities, covering over 60% of the population, were active in ‘Efsharbari in the City’ and received funding for health promotion activities (Table 2). Moreover, there was an increase in participating municipalities representing lower socio-economic clusters and minority populations.

**Fig. 3:**
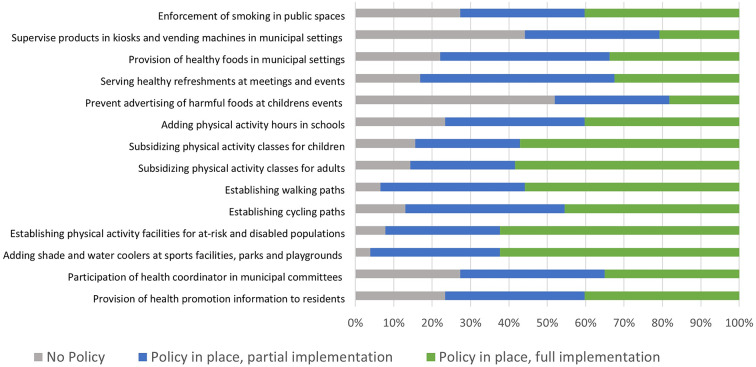
Implementation of different health promotion policies in ‘Efsharibari in the City’, 2022, N = 77

**Fig. 4:**
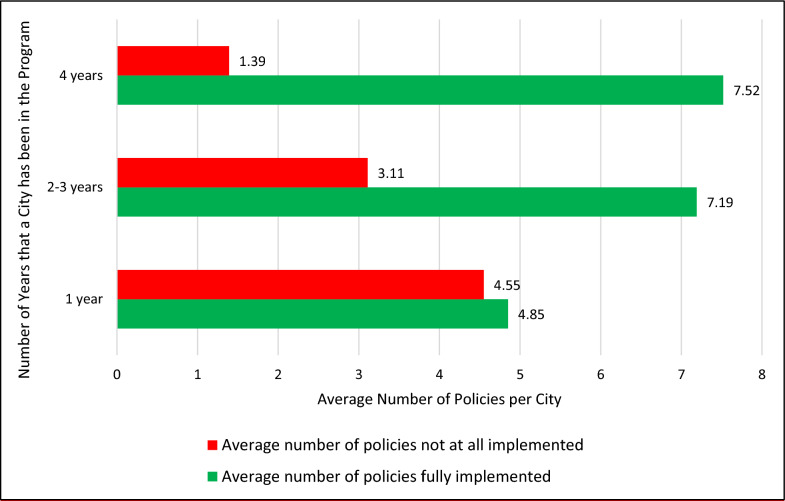
Policy implementation level in ‘Efsharibari in the City’ municipalities by years of experience

A survey was conducted annually for all participating cities, during the grant cycle of ‘Efsharibari in the City.’ It examines key health promotion indicators including collaboration processes, adoption of health policies and community participation [21, 22]. The 2022 survey compared municipalities previously in the system to new grantees. It shows that as municipalities gain experience they increase collaborative programs within and outside the municipality and they program with more diverse populations. The 2022 survey also showed that municipalities have been quicker to implement physical activity policies, in particular related to changes to the environment such as establishing facilities and walking paths, but slower to implement policies related to provision of healthy foods or smoking enforcement (Fig. 3). As municipalities gain experience they fully implement more policies (Fig. 4). Among the ‘Efsharibari in the City’ municipalities, about 60% reported to the Minister of Health on enforcement of smoking laws, compared to 18% of the non-participating municipalities [23].

The Ministries of Health and Education set a common goal to promote health across the entire school system as well as to initiate and expand a network of Health Promoting Schools, based on the WHO Global School Health Initiative. Various health promotion policies have been introduced. They include adding 90 min of physical activity per week, mandatory physical education classes and at least two sessions of nutritional education per year. The number of schools offering expanded matriculation in sports doubled. There was an increase in the percentage of 5th graders provided with swimming instruction (from 40 to 60% nationally) (Table 2). Educational tools, training, and programs were developed for preschool through secondary education focusing on an active and healthy lifestyle – throughout the year as well as on holidays and special occasions.

**Fig. 5:**
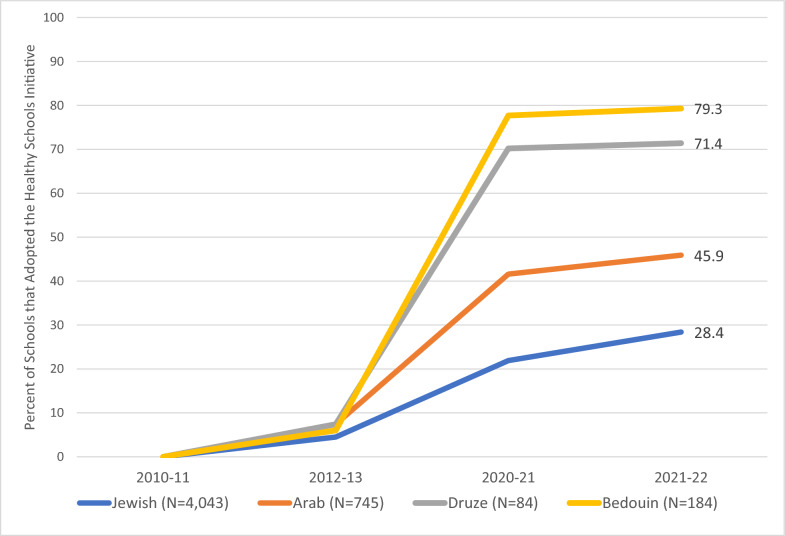
Change in percent of Health Promoting Schools, by Ethnicity, 2010–2022

During the decade, over a third of primary and secondary state schools joined the Health Promoting Schools network (Table 2). Recognized health-promoting schools in Israel commit to adding health-related policies and programming and received a small grant. While there was a substantial increase in health promoting-schools nationwide, the increase was higher in areas with minority populations (Arabs 46%, Druze 71%, and Bedouins 79%) than in the Jewish population (28%) (Fig. 5). Consistent with these measures, 70% of students in grades 5–6 and 35% of the students in grades 10–11 reported, in a recent national survey, that their school encourages active and healthy living – with higher rates for students in the Arab school track (Rama, 2022).

### Social marketing

One of the objectives of Efsharibari is to advance social norms that support active and healthy living in Israel. A key strategy for the development of social norms is communication and education. The social marketing approach adopted for this purpose focused on three key areas: brand implementation, digital media and campaigns [24, 25]. Creation of the Efsharibari brand name, branding and design language took place in 2014 as a coordinated effort between the inter-ministerial committee and Tel Aviv University [26]. The name Efsharibari ("A healthy lifestyle is possible") embodies a national responsibility to support an active and healthy lifestyle. A uniform branding language was established in terms of messages, content, graphics, and photographs, which was then used to develop several sub-brands, such as ‘Efsharibari in the City’, ‘Efsharibari at Work’ and ‘Efsharibari Breastfeeding’. Surveys conducted in 2011 and 2015 exhibit increases in awareness of the program and the brand (19% and 7.8% respectively); however, there have been no additional surveys of public awareness since 2015 [24].

Targeted digital media channels were developed for Efsharibari in accordance with the social marketing approach. These included a website, Facebook page, Instagram profile, Twitter-X profile and YouTube channel. The Efsharibari website is a leading national content hub for professional health promotion. It has files available for download, production and printing, as well as videos and presentations. There are also professional how-to guides for municipalities, workplaces and schools. A secondary audience is the general public; therefore, the website provides information in clear and simple language. In 2019, a new website was designed for the MOH cloud, adapted for mobile browsing and accessible to people with disabilities. Subsequently, site content search on Google has improved, leading to a 100% increase in the use of the site (+ 153,554 users) and an increase in viewed webpages (+ 332,603 viewed). In 2022 the website was translated to Arabic and English. It is important to note that with the exception of a few YouTube videos in Arabic, the other digital platforms besides the website, are available only in Hebrew. The YouTube channel is comprised of 111 videos in different languages. There have been 1,214,244 viewers as of 2022. Content for the digital channels targets various areas of interest and is updated in accordance with national events.

During the decade of action, Efsharibari launched a number of media campaigns to promote a healthy and active lifestyle. In accordance with the social marketing approach, several campaigns were segmented for different target audiences such as parents of young children or for different minority groups such as the Arab or Ultra-Orthodox Jewish communities. Campaigns were typically launched prior to or during a change in policy and or in support of intervention programming; several campaigns won awards. The ‘Efsharibari Birthday Party’ (October 2014), adopted by the Ministry of Education, promoted healthier snacks in preschool parties. ‘Sweetened Beverages: Not in our Home’ (August 2017) promoted social norms of serving water while hosting. ‘Red—Stop, Green – Go’ (December 2019) was an information campaign to support the FOP labeling process; ‘National Walking Day: Every Minute Counts’ (annually November 2014–2022) promoted the value of daily physical activity; coinciding with World Walking Day, it was conducted at hundreds of events, nationwide, each year.

### Health behaviors and morbidity

Health behavior and morbidity indicators, representing national changes in health indices related to a variety of influences in the environment, were included in the indicator framework as output and outcome measures for Efsharibari activities. Healthy behaviors are short- and middle-term outputs and outcomes. They can be associated with individual health and they can also be linked to changes in policies and the enabling environment, which are the core of Efsharibari activities [27–29]. The findings for health behaviors are based on various surveys with representative samples of the population in Israel; the frequency and scope of health behavior surveys increased during the years of Efsharibari activity. Findings for nutrition and physical-activity behaviors are mixed (See Table 3). Exclusive breastfeeding saw the most substantial reductions, in particular among Arab women and infants (e.g., a 24% reduction from 2014 to 2012 in breastfeeding by one–month-old babies, whereas the reduction was 10% for Jewish women and infants). There were improvements in reported consumption of healthy foods for both adolescents and adults, such as eating more vegetables and reducing sweet drinks or sugar. However, there were declines in healthy dining habits: fewer family meals and adolescents are less likely to eat breakfast.

**Table 3:**
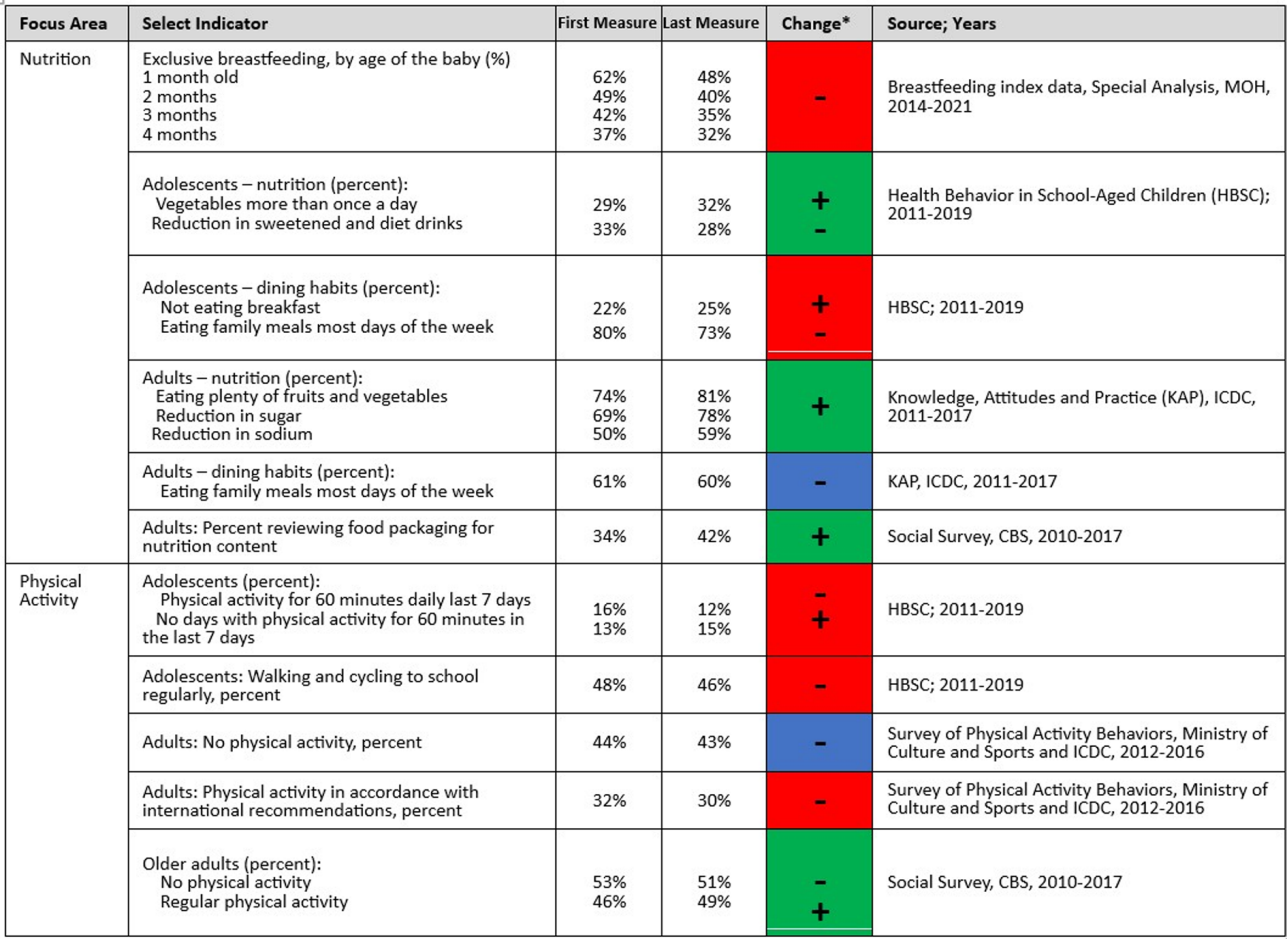
Changes in health behaviors

* Green indicates positive change (over 2 percentage points or equivalent), red indicates negative change (over 2 percentage points or equivalent), blue indicates little change (less than 2 percentage points or equivalent), grey indicates missing information; the ± presents the direction of the findings

Physical activity indicators were tracked for adolescents (12 to 18 years old), adults (20 to 64), and seniors (65 and older). These indicators are the rates of compliance with international recommendations for physical activity for each age group. Adolescents report declines in various physical activity measures. Moreover, their rate of physical activity decreases with age. The rate of physical activity among girls is significantly lower (about half) than that of boys in all the adolescent years. Physical activity measures declined to some extent for adults 20 to 64 years old. Seniors, however, presented modest increases in physical activity including strengthening exercises.

**Fig. 6:**
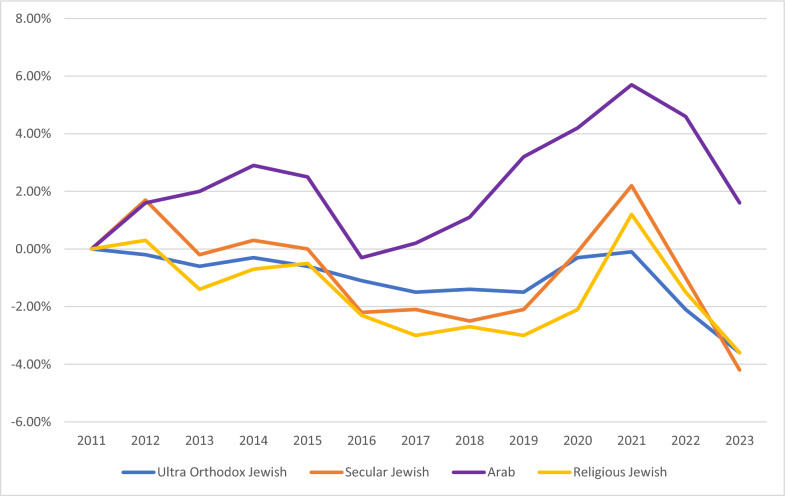
First grade children, proportion overweight and obese, percentage point change since 2011 by school type

**Fig. 7:**
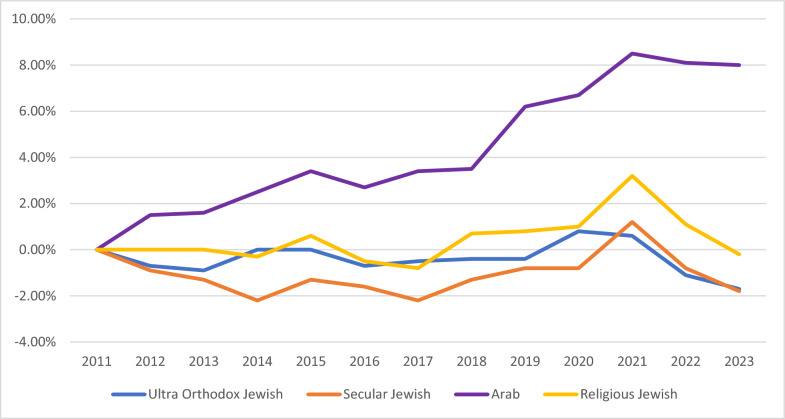
Seventh grade children, proportion overweight and obese, percentage point change since 2011 by school type

Morbidity and mortality, including data on obesity and NCDs, serve as long-term outcome indicators – with the understanding that these measures are also influenced by other factors, such as healthcare quality (See Table 4). When the National Student Health Service of the MOH conducts vaccinations and health education at schools, they also measures pupils’ height and weight. Overall, there have been moderate increases in the percent of children (1st grade) and teenagers (7th grade) who are overweight or obese. However, this trend is substantially higher for the Arab/Bedouin populations. In a review of trends for students in first grade across different school tracks – Jewish (unaffiliated), Religious Jewish, Ultra-Orthodox Jewish, and Arab – there has been a decrease for the three Jewish school tracks but an increase in the Arab schools for most years (Fig. 6). In the seventh grade, there were mixed trends for the Jewish school tracks, which saw slight decreases and increases over the period, while the Arab school tracks had an increase in overweight and obese adolescents during this period (Fig. 7). The years of the COVID-19 pandemic, 2020 and 2021, during which a quarantine policy was in effect, were characterized by an increase in obesity among students in first and seventh grade, in all school tracks.

**Table 4:**
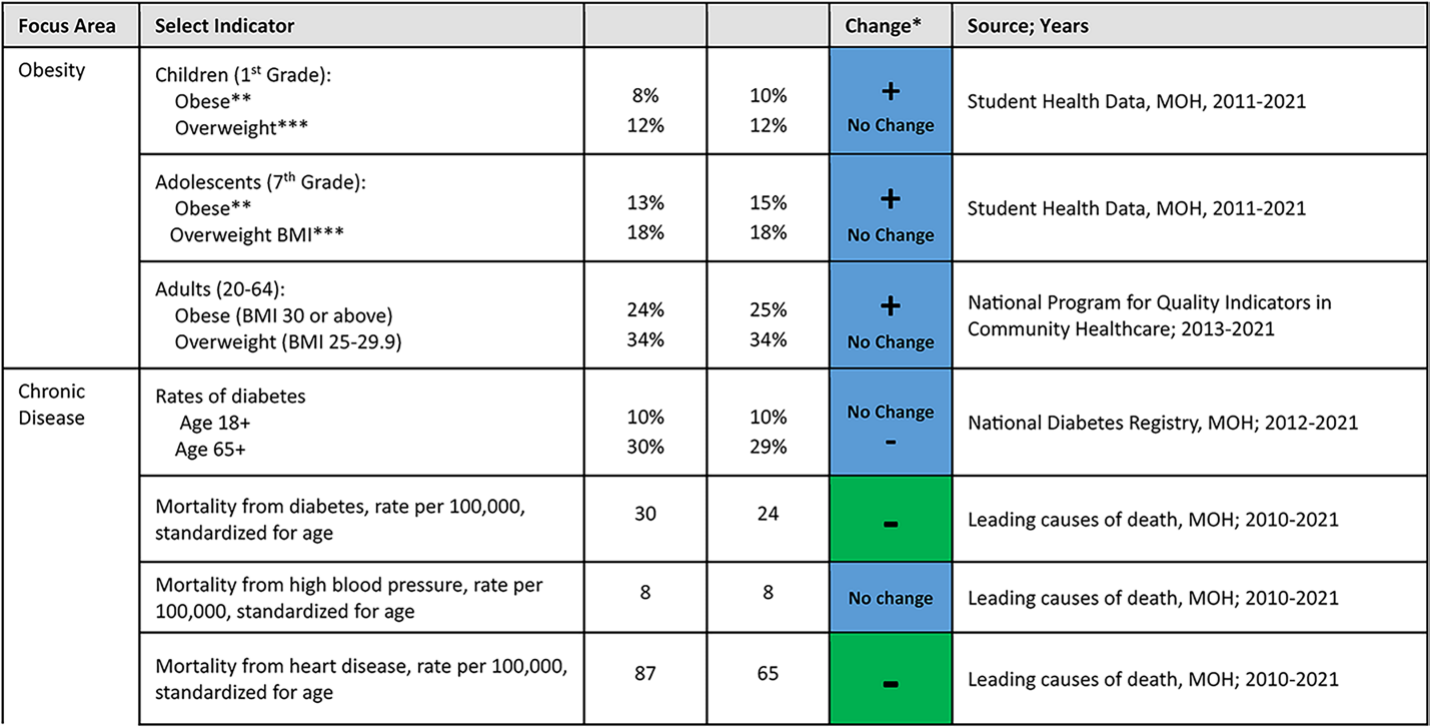
Changes in morbidity and mortality^1^

1 All data presented in the table is collected for the entire population, not samples

In 2013, the National Program for Quality Indicators in Community Healthcare began to collect height and weight data among adults in all of the national health maintenance organization (HMO) networks. These indicators make it possible to track trends and changes in obesity rates among adults in Israel. Between the years 2013 and 2021, there was a slight increase in obesity rates. In 2021, the overall rate of overweight and obese adults was 59.4 percent. The prevalence of obesity is higher among men and women in a lower socioeconomic status.

Table 4 shows substantial overall reductions from 2010 to 2021 in death rates from diabetes and heart disease, although the incidence of diabetes has changed little. The incidence of diabetes and heart disease is higher in the Arab population than in the Jewish population. In all age groups, the highest rates of diabetes are among the Bedouin and Arab population, compared to the Jewish population and the Ultra-Orthodox Jewish population. Also, in most age groups, the diabetes rates in the Ultra-Orthodox Jewish population were higher than in the rest of the Jewish population [30].

## Discussion

The Israel National Program for Active and Healthy Living–Efsharibari was launched at the end of 2011, with the aim of creating an environment and settings that enable and foster social norms in support of a healthy lifestyle [13]. Efsharibari was developed based on core principles of health promotion; its evaluation model, indicator framework, and activity domains are based on international recommendations, tailored to a national setting [4, 12, 17]. A review of key objectives and indicators over the first decade of action serves to identify achievements and highlight gaps that may be targeted in the next phases of the program.

At the policy and planning level, Efsharibari implemented many principles of the HiAP model. These include promotion of interconnected policies on healthy nutrition and physical activity that work together to support a healthy lifestyle [31, 32]. These principles are reflected in achievements, such as the establishment of a systemic national plan, regulatory processes, the use of economic tools, and collaborative efforts. Of particular note is the inter-ministerial steering committee, which remained viable for over a decade and brought about changes in health promotion. This continuity was achieved despite political instability, the lack of a fixed budget, and the disruptions during the COVID-19 pandemic. This success reflects the power of inter-sectoral or cross-government professional teams in designing and promoting policies for active and healthy living "from the middle out" [33–35]. Findings also point to progress in the specific policy goals, based on recognized “best practices” for preventing NCDs and for promoting healthy nutrition and physical activity with a relatively high ranking (28 out of 151 countries) [36, 37]. Correspondingly, in 2022 an inter-ministerial committee for ‘Health promotion and the prevention of NCDs’ was established, including partners from different government agencies. The committee is headed by the MOH director general and managed by the public health and strategic and economic planning divisions of the MOH. The committee published its recommendations, emphasizing the need for permanent and significant investment in prevention as well as the need for further inter-ministerial and inter-systemic partnerships [38].

Efsharibari promoted a settings-based approach, establishing frameworks that promote health, primarily in municipalities, educational institutions, and workplaces. This approach is grounded in the understanding that the environment and the organizations in which an individual spends a significant amount of time have an impact on health [39]. The efforts included development of policies, procedures, toolkits and training for each environment and target audience. Promotion of health in municipalities demanded a paradigm shift in the MOH, the Ministry of the Interior, the Federation of Local Authorities and even among the general public in Israel, none of whom had previously viewed health as the purview of municipalities. Moreover, understanding has increased that social determinants of health, behaviors and lifestyle have a significant impact on health in local and national settings [10, 11]. The COVID-19 pandemic further emphasized the ability and interest of municipalities to care for the health of their residents [40]. The ‘Efsharibari in the City’ model proved effective in increasing municipality commitment to developing infrastructure for health promotion and increasing the number of cities participating in the Healthy Cities Network. A recent evaluation found that the Health Cities Network in Israel continues to be a robust participant in the global WHO initiative, sharing the same fundamental goals and complying with similar requirements as European cities with the exception of conducting periodic health profiles [41]. In the education system, the collaboration between the MOH and Ministry of Education in Efsharibari resulted in a commitment to increase health of children in Israel via joint objectives, policies, and programs – especially, the Health Promoting Schools network. While the increases in health-promoting municipalities and schools are promising, it is necessary to continue to develop benchmarks, programs and tools to change health behaviors. Health-promoting settings may be further anchored in regulatory policy to support allocation of manpower and resources.

Efsharibari developed a platform and digital assets for reliable information, content and tools. They integrate messages for a variety of audiences in a unique health-promotion language. A particular emphasis was placed on social marketing strategies to support positive social norms and habits among the public and within different organizations (e.g., healthy birthdays, breastfeeding) [25, 42–44]. The efforts to develop supporting environments have also resulted in a substantial increase in the number of stakeholders involved in health promotion–in the education system, municipalities, media, workplaces and government agencies. Overall, these measures seem to reflect a positive trend and progress in awareness and activities related to a healthy lifestyle in Israel during the first decade of Efsharibari.

Improved nutrition in Israel is reflected in the healthier nutritional intake in the daily diet, and in the provision of healthier foods in different settings, especially in schools. The food industry experienced notable changes through voluntary reformulation processes and nutritional labeling regulations. Already in the first years of Efsharibari, even prior to the FOP labeling reform, collaboration with the food industry achieved a significant reduction in the amounts of sodium in basic products such as bread and cheese. The FOP labeling further promotes production of healthier products and encourages healthier food choices and Israel was one of the early adopters of mandatory FOP labeling, the first in Europe [45]. Both adults and adolescents reported positive trends in nutrition behaviors. Lower consumption of sodium, saturated fats and sugars were found in both the adult and adolescent diet. The tax on sweetened beverages contributes to health equity because it most affects the low socioeconomic population, which is usually at higher risk [29, 46]. The tax coincided with campaigns promoting the drinking of water rather than sweetened beverages. This demonstrated the importance of adding chilled water facilities in public places [47]. Following completion of the evaluation study, the tax on sweetened beverages was cancelled after a change in the government, which potentially contributed to harming public confidence as well as public health [48]. Continued progress in nutrition likely entails maintaining and enhancing the FOP labeling, further reducing advertising and access to harmful foods such as reintroduction of the taxation on sweetened beverages, while simultaneously increasing access to healthy choices – e.g., by reducing costs for fruits and vegetables [49].

There has been a notable decrease in exclusive breastfeeding of infants, in particular among Arab women. A decrease in breastfeeding patterns is related to employment rates, conditions in workplaces, barriers related to breastfeeding in public, social norms and more [50, 51]. Future intervention activities may focus on enabling environments for breastfeeding as well as further individual training to support breastfeeding with an emphasis on populations that have recently increased rates of employment outside the home, especially among Arab and Ultra-Orthodox Jewish women.

Studies show that increased urbanization and rapid economic development are associated with a reduction in physical activity and an increase in screen use – in contrast with the goal of achieving a more active lifestyle, as promoted in the national program [52–54]. Israel is experiencing increased population density, with a high percentage of residents in urban and suburban municipalities [55]. Research points to an association of the built environment and physical activity, especially on the use of active transport [56, 57]. In the last decade, national and local government and other key players in Israel have improved access to sports facilities, parks, active travel routes and they have expanded activities in the community. Nevertheless, there is still a need for improvement, including reducing gaps between different populations in Israel such as between the Arab and Jewish communities and between the general population and Ultra-Orthodox Jewish communities [58, 59].

Trends in reported physical activity behaviors show moderate change. There has been a decrease in the proportion of adults and teenagers who report participation in physical activity in accordance with international recommendations, whereas seniors over 65 reported some improvement in physical activity behaviors. Physical activity among adults is lower in Israel (30% in 2016) compared to the average in OECD countries (40% in 2019 respectively) [60]. Moreover, the Arab population is less physically active than the Jewish population and men are more active than women. A decline in physical activity has been reported for children and adolescents worldwide [61, 62]. In Israel, there is a decrease in physical activity rates with age, especially in the transition from elementary school to secondary school. Girls report approximately half of the physical activity as compared to boys [63]. Israel’s National Report Card for Child and Adolescent Physical Activity, conducted as part of the Global Matrix 4.0 which examined 10 leading indicators of physical activity, pointed to an overall physical activity score of D- and an average score of D + on the individual indicators. For these indicators, the percent of children and teenagers in Israel meeting the recommended level ranged from just 20 to 39 percent [64, 65]. These scores were worse than any of the 20 European countries that participated in the international measurement. In early stages of the program, Efsharibari adopted the approach “Every minute counts” to encourage physical activity in daily activities, with the aim of promoting active living also among the population that is not traditionally engaged in sports. In light of the disparities in physical activity rates by age, gender and sector as well as the generally low participation in physical activity, future intervention measures must target unique needs of those populations that are not engaged as well as increasing physical activity among more active populations. Promotion of social norms and environments that allow for incorporating physical activity into daily living, during both leisure and work hours, have the potential to support active and healthy living [66, 67]. Increased awareness of the contribution of physical activity and sports to psychosocial aspects at the personal and community level as well as to physical and emotional resilience has the potential to support more extensive investment in this area [68–70].

Morbidity and mortality trends further demonstrate gaps between populations in Israel. Obesity trends for children and adolescents point to higher increases for Arab and Bedouin students as compared to Jewish students. It is important to examine these trends in light of the food environment and availability of healthy choices as well as to develop tailored interventions for at-risk populations. In comparison to international trends, the rate of children with obesity in Israel is one of the lowest compared to most countries in the European region [71]. Moreover, Israel’s ranking in relation to OECD countries has improved over the years. Among adults, the prevalence of obesity and overweight remained relatively similar between 2013 and 2019, in contrast to a general trend of increase in most high-income countries [72]. In most OECD countries with available data, there have been consistent increases in overweight and obesity rates of 1 to 2 percentage points every year [60]. Data on NCDs demonstrate significant decreases over time in mortality rates from heart disease and diabetes. Disparities between population groups are noticeable, as well as on the basis of socio-economic rank, in particular for diabetes and obesity [58, 73].

The current study reviewed a framework of indicators to describe trends over a decade, however there are limitations in the current design. A multilevel statistical model was not utilized to analyze the association between the program and health outcomes. This review of trends over time is descriptive in nature. Therefore, associations are limited and ecological.

There has been progress in data systems during the first decade of Efsharibari – specifically, in national registries, such as the capture of height and weight data via the Student Health Registry, the Diabetes Registry, and the National Program for Quality Indicators in Community Healthcare Data. Data on health behaviors, however, are primarily collected via self-reported surveys. Although these surveys have improved significantly, they remain limited in scope, consistency and reliability.

Data sources need to be interpreted with caution. For example, as indicated above, data on household food expenditure may reflect changes in the cost of living rather than changes in consumption. In addition, several data sources are based on self-reporting, which may introduce bias to the findings.

Finally, the health behaviors and morbidity rates that serve as output and outcome measures are not influenced solely by Efsharibari activities; they are related to multiple factors including healthcare quality. As behavior change is linked to long-term changes in health, it is important to acknowledge these limitations.

Investment in the collection and analysis of data at the national and local level is recommended. That includes systematic measures via well-baby clinics, student health services and the education system, and HMO data. Potentially, that could be enhanced by longitudinal studies in a broad and representative samples of the population. Innovative technological methods for data collection and analysis may also be considered, such as large-scale data collection on physical activity from digital and cellular applications that have been adopted across the different HMOs in Israel. It is important to update the Efsharibari indicators for nutrition and physical activity in accordance with the current findings and develop indicators for the newer focus areas such as smoking and stress reduction. Future intervention and evaluation activities will need to improve study of gaps and at-risk populations and incorporate further data on social determinants of health. Future evaluation studies may incorporate stronger methods to determine causality using comparison groups or multi-level modeling. Finally, evaluation processes that include additional studies of specific intervention programs within the national Efsharibari framework are important. Research indicates that the attempt to assess the impact of national programs using indicators at the level of the general population is limited [74]. Studies to evaluate the effectiveness of specific efforts within the framework of the national program can provide information regarding the contribution and the limitations of various interventions, as well as allow for updates and adjustments in real time that could enhance program efficacy [75].

## Conclusions

The Israel National Program for Active and Healthy Living–Efsharibari was designed in accordance with international recommendations, to promote the health of the population and prevent NCDs [4, 20]. The first decade of action demonstrates important achievements for indicators in the key activity domains and in accordance with the initial program goals: promoting healthy nutrition and physical activity; and fostering leadership, advocacy and the promotion of national and local infrastructures and settings that enable active and healthy living. In the coming decade, enforcement, process improvement, and implementation of new initiatives while continuing ongoing activities, serve as complex challenges.

The importance of health and well–being as a significant component of national and individual resilience, as seen in the COVID-19 pandemic, wars and times of crisis in Israel and worldwide, should inspire future efforts in Efsharibari. Specifically, inclusion of physical activity and healthy nutrition in all programs related to well-being, resilience and mental health is imperative due to their contribution to stress reduction. Moreover, health organizations worldwide recommend formulating preparedness plans to address potential harm to health service access, physical activity, healthy nutrition and healthy lifestyles in emergency situations (epidemics, natural disasters, wars).

The unequal incidence of NCDs among different population groups in Israel is an urgent call for greater engagement in the national program. That includes support for both national and local efforts to cope with issues such as food insecurity, unequal access to safe public green spaces, exposure to working conditions harmful to health, loneliness, stress and anxiety. An ongoing commitment including allocation of resources and manpower in accordance with updated objectives is recommended. So are stronger evaluation processes focusing on understanding causality. Inter-ministerial planning and coordination are necessary to develop health promotion policies for the next decade based, especially addressing issued identified in this review: ongoing gaps in health behaviors and health disparities between population groups. They should address additional activity domains, such as smoking and stress reduction. As new health risks arise, the multidisciplinary approach developed in Efsharibari, will be needed to promptly identify and pro-actively provide solutions tailored to Israeli culture and society.

## Supplementary Information

Additional file 1

## Data Availability

The datasets used and/or analyzed during the current study are available in the public domain or from the corresponding author on reasonable request.

## Abbreviations

CBS: Central bureau of statistics
FOP: Front of package (Labeling)
HBSC: Health behavior in school-age children
HiAP: Health in all policies
HMO: Health maintenance organization
ICDC: Israeli center for disease control
KAP: Knowledge, attitudes and practice
MOH: Ministry of health
NCDs: Noncommunicable diseases
OECD: Organisation for economic co-operation and development
WHO: World health organization
