# Supplementary material for: Reflecting on a decade of action: a review of select indicators for the Israel National Program for Active and Healthy Living–Efsharibari, 2011–2022
Source: Isr J Health Policy Res. 2025 Oct 22;14:60. doi: 10.1186/s13584-025-00722-3 (PMC12542037; doi:10.1186/s13584-025-00722-3)
Supplement: Supplementary file 1 — Additional file 1 [file 13584_2025_722_MOESM1_ESM.docx]

Supplemental Table: Schedule of Measurements included in the Indicator Framework

| Domain | Indicator/ Indicator Set | Source | Years Collected | Review in Indicator Framework |
| --- | --- | --- | --- | --- |
| Policy | Policy Domain Indicators | Interviews with Policymakers and Document Review | 2011, 2015, 2022 | Research Team |
| Supporting Environment | Measures of Sodium in Typical Daily Diet | MABAT Surveys, Israel Center for Disease Control (ICDC) | 2011, 2013, 2015, 2017, 2022 | Nutrition Division, Research Team |
|  | Measures of Saturated Fats and Sugar in Typical Daily Diet | MABAT Surveys, ICDC | 2017, 2022 | Nutrition Division, Research Team |
|  | Average monthly household expenditure on products | Household Expenditure Survey, Central Bureau of Statistics (CBS) | 2011, 2013, 2014, 2018, 2019 | Research Team |
|  | Caloric value available to the population; Key foods available to the public | Food Supply Data, CBS | 2011, 2012, 2013, 2014, 2019, 2020, 2021 | Research Team |
|  | Availability of safe places to walk in the dark | Social Survey, CBS | 2010, 2013, 2015, 2017, 2020 | Research Team |
|  | Satisfaction with green spaces, public gardens/parks in community | Social Survey, CBS | 2011, 2013, 2015, 2018, 2022 | Research Team |
|  | Access to publicly available gymnasiums in the afternoon | Survey of Physical Activity Behaviors, ICDC | 2012, 2016 | Research Team |
|  | Public facilities: gymnasiums/ sports fields/ outdoor sports equipment/ other | Facilities Records, Ministry of Culture and Sports | 2009, 2022 | Research Team |
|  | Local authorities in Efsharibari in the City | Efsharibari, Ministry of Health (MOH) | 2010, 2013, 2018, 2019, 2020, 2022 | Research Team |
|  | Local authorities participating in the WHO Healthy Cities Network | Healthy Cities Network | 2010, 2013, 2018, 2019, 2020, 2022 | Research Team |
|  | Health Promoting Schools | Health Unit, Pedagogical Division, Ministry of Education | 2010, 2012, 2014, 2020, 2021 | Research Team |
|  | Expanded sports matriculation | Commissioner of Physical Education, Ministry of Education | 2010, 2012, 2014, 2020 | Research Team |
|  | Students in grade 5 provided with swimming instruction | Commissioner of Physical Education, Ministry of Education | 2010, 2020 | Research Team |
| Social Marketing | Awareness of Brand | Efsharibari, MOH | 2011, 2015 | Research Team |
|  | Website Access | Efsharibari, MOH | 2011, 2015, 2018, 2020, 2022 | Research Team |
|  | Social Media Channel Access | Efsharibari, MOH | 2011, 2015, 2021, 2022 | Research Team |
|  | National Media Campaigns | Efsharibari, MOH | 2014-2022, Annually | Research Team |
| Health Behaviors and Morbidity | Exclusive breastfeeding | Breastfeeding index data, MOH | 2014, 2018, 2021 | Mother, Child & Adolescent Department, Research Team |
|  | Adolescents: Health Behaviors – Nutrition, Physical Activity | Health Behavior in School Age Children, Bar Ilan University | 2011, 2014, 2019 | Research Team |
|  | Adults: Eating plenty of fruits and vegetables; Reduction in sugar / sodium; Eating family meals | Knowledge, Attitudes and Practice (KAP), ICDC | 2011, 2013, 2017 | Research Team |
|  | Adults: Reviewing food packaging for nutrition content, percent | Social Survey, CBS | 2010, 2017 | Research Team |
|  | Adults: No physical activity, Physical activity in accordance with international recommendations | Survey of Physical Activity Behaviors, ICDC | 2012, 2016 | Research Team |
|  | Older adults: No physical activity / Regular physical activity | Social Survey, CBS | 2010, 2017 | Research Team |
|  | Child and Adolescent: Measures of BMI | Student Health Data, MOH | 2011, 2012, 2013, 2014, 2015, 2021 | Research Team |
|  | Adults: Measures of BMI | National Program for Quality Indicators in Community Healthcare | 2013, 2015, 2021 | Research Team |
|  | Adults: Rates of Diabetes | National Diabetes Registry, MOH | 2012, 2013, 2015, 2016, 2018, 2020, 2021 | Research Team |
|  | Adults: Mortality from diabetes, high blood pressure, heart disease | Leading Causes of Death, MOH | 2010, 2013, 2015, 2017, 2018, 2020, 2021 | Research Team |
